# Supplementary material for: Post-Diagnosis Vitamin D Supplement Use and Survival among Cancer Patients: A Meta-Analysis
Source: Nutrients. 2022 Aug 19;14(16):3418. doi: 10.3390/nu14163418 (PMC9413994; doi:10.3390/nu14163418)
Supplement: Supplementary file 1 [file nutrients-14-03418-s001.zip › nutrients-1854932-SI.pdf]

Table S1. PRISMA Guideline

Section/topic	#	Checklist item	Reported on page #
**TITLE**			
Title	1	Identify the report as a systematic review, meta-analysis, or both.	1
**ABSTRACT**			
Structured summary	2	Provide a structured summary including, as applicable: background; objectives; data sources; study eligibility criteria, participants, and interventions; study appraisal and synthesis methods; results; limitations; conclusions and implications of key findings; systematic review registration number.	1
**INTRODUCTION**			
Rationale	3	Describe the rationale for the review in the context of what is already known.	1
Objectives	4	Provide an explicit statement of questions being addressed with reference to participants, interventions, comparisons, outcomes, and study design (PICOS).	2
**METHODS**			
Protocol and registration	5	Indicate if a review protocol exists, if and where it can be accessed (e.g., Web address), and, if available, provide registration information including registration number.	2
Eligibility criteria	6	Specify study characteristics (e.g., PICOS, length of follow-up) and report characteristics (e.g., years considered, language, publication status) used as criteria for eligibility, giving rationale.	2-3
Information sources	7	Describe all information sources (e.g., databases with dates of coverage, contact with study authors to identify additional studies) in the search and date last searched.	2-3
Search	8	Present full electronic search strategy for at least one database, including any limits used, such that it could be repeated.	Table S2
Study selection	9	State the process for selecting studies (i.e., screening, eligibility, included in systematic review, and, if applicable, included in the meta-analysis).	2

Data collection process	10	Describe method of data extraction from reports (e.g., piloted forms, independently, in duplicate) and any processes for obtaining and confirming data from investigators.	3
Data items	11	List and define all variables for which data were sought (e.g., PICOS, funding sources) and any assumptions and simplifications made.	3
Risk of bias in individual studies	12	Describe methods used for assessing risk of bias of individual studies (including specification of whether this was done at the study or outcome level), and how this information is to be used in any data synthesis.	3-4
Summary measures	13	State the principal summary measures (e.g., risk ratio, difference in means).	3-4
Synthesis of results	14	Describe the methods of handling data and combining results of studies, if done, including measures of consistency (e.g., I^2) for each meta-analysis.	3-4

**Section/topic**	**#**	**Checklist item**	**Reported on page #**
Risk of bias across studies	15	Specify any assessment of risk of bias that may affect the cumulative evidence (e.g., publication bias, selective reporting within studies).	3-4
Additional analyses	16	Describe methods of additional analyses (e.g., sensitivity or subgroup analyses, meta-regression), if done, indicating which were pre-specified.	3-4
**RESULTS**			
Study selection	17	Give numbers of studies screened, assessed for eligibility, and included in the review, with reasons for exclusions at each stage, ideally with a flow diagram.	4<br>Figure 1
Study characteristics	18	For each study, present characteristics for which data were extracted (e.g., study size, PICOS, follow-up period) and provide the citations.	Table S3<br>Table S4
Risk of bias within studies	19	Present data on risk of bias of each study and, if available, any outcome level assessment (see item 12).	Table S3

Results of individual studies	20	For all outcomes considered (benefits or harms), present, for each study: (a) simple summary data for each intervention group (b) effect estimates and confidence intervals, ideally with a forest plot.	Table S3<br>Figure 2-4
Synthesis of results	21	Present results of each meta-analysis done, including confidence intervals and measures of consistency.	4-11<br>Figure 2-4
Risk of bias across studies	22	Present results of any assessment of risk of bias across studies (see Item 15).	4-11
Additional analysis	23	Give results of additional analyses, if done (e.g., sensitivity or subgroup analyses, meta-regression [see Item 16]).	4-11
**DISCUSSION**			
Summary of evidence	24	Summarize the main findings including the strength of evidence for each main outcome; consider their relevance to key groups (e.g., healthcare providers, users, and policy makers).	11-12
Limitations	25	Discuss limitations at study and outcome level (e.g., risk of bias), and at review-level (e.g., incomplete retrieval of identified research, reporting bias).	12
Conclusions	26	Provide a general interpretation of the results in the context of other evidence, and implications for future research.	12
**FUNDING**			
Funding	27	Describe sources of funding for the systematic review and other support (e.g., supply of data); role of funders for the systematic review.	13

From: Moher D, Liberati A, Tetzlaff J, Altman DG, The PRISMA Group (2009). Preferred Reporting Items for Systematic Reviews and Meta-Analyses: The PRISMA Statement. PLoS Med 6(6): e1000097. doi:10.1371/journal.pmed1000097

For more information, visit: **www.prisma-statement.org**.

Table S2. Search Terms

**Pubmed**	("Vit D"[Mesh] OR Vit D[tw] OR cholecalciferol*[tw] OR ergocalciferol*[tw] OR “calcium”[Mesh] OR calcium[tw]) AND (“dietary supplements”[Mesh] OR supplement[tw] OR supplements[tw] OR supplementation[tw]) AND ("neoplasms"[Mesh] OR neoplasm[tw] OR neoplasms[tw] OR neoplasia[tw] OR cancer[tw] OR cancers[tw] OR tumor[tw] OR tumors[tw] OR tumour[tw] OR tumours[tw] OR carcinoma[tw] OR carcinomas[tw]) AND (“mortality”[Mesh] OR mortality[tw] OR death[tw] OR survival[tw]) NOT (Case Reports[ptyp] OR Comment[ptyp] OR Letter[ptyp] OR Editorial[ptyp]) NOT ("animals"[Mesh] NOT "humans"[Mesh])
**Embase**	('Vit d'/exp OR ‘Vit D’:ab,ti OR cholecalciferol:ab,ti OR ergocalciferol:ab,ti OR 'calcium'/exp OR calcium:ab,ti) AND (‘dietary supplements’/exp OR supplement:ab,ti OR supplements:ab,ti OR supplementation:ab,ti) AND ('neoplasms'/exp OR neoplasm:ab,ti OR neoplasms:ab,ti OR neoplasia:ab,ti OR cancer:ab,ti OR cancers:ab,ti OR tumor:ab,ti OR tumors:ab,ti OR tumour:ab,ti OR tumours:ab,ti OR carcinoma:ab,ti OR carcinomas:ab,ti) AND (‘mortality’/exp OR mortality:ab,ti OR death:ab,ti OR survival:ab,ti) NOT ('case report'/de OR ‘comment’/de OR ‘letter’/de OR 'editorial'/de) NOT ([animals]/lim NOT [humans]/lim)

Table S3. Main Characteristics of Studies Included

First author, Year, Country, Reference	Trial name<br>Cancer patients	Age<br>Male (%)<br>Follow-up period	Timing of Vit D supplementation<br>Contrast for RR	Overall Survival: RR (95% CI)	Progression-free Survival: RR (95% CI)	Cancer-specific Survival: RR (95% CI)	Relapse: RR (95% CI)	Exclusion criteria regarding Vit D supplementation
**Randomized Controlled Trials**								
Urashima 2019 Japan [14]	AMATERASU<br>digestive tract cancers (I-III)	66<br>66%<br>median: 3.5 y	2-4 wk after operation<br>Vit D3(2000 IU/d) vs. placebo	0.81 (0.48, 1.36)	0.66 (0.43, 0.99)	1.09 (0.58, 2.01)	0.75 (0.48, 1.17)	Excluded current users taking Vit D supplement or active Vit D
Ng 2019 US [9]	SUNSHINE<br>advanced or metastatic colorectal cancer	56<br>57%<br>median: 1.9 y	during chemotherapy<br>Vit D3(8000/4000IU/d) vs. 400 IU/d	NA	0.64 (0.43, 0.96)	NA	NA	Excluded current users of taking 2000 IU/d or greater of Vit D3
Akiba 2018 Japan [11]	UMIN000001869<br>Non-small cell lung cancer	68<br>62%<br>median: 3.3 y	after operation (during chemotherapy)<br>Vit D3(1200 IU/d) vs. placebo	1.22 (0.54, 2.79)	1.15 (0.64, 2.05)	NA	NA	Excluded current users taking Vit D supplement or active Vit D

	stage IA to IIIA							
Johansson 2021 Italy [13]	N. EudraCT 2009-012049-46 recently resected, stage II melanoma\	50.5 57% 1 y	after operation (no adjuvant treatment) Vit D3(100,000 IU/50d) vs. placebo	NA	1.20 (0.37, 4.00)	NA	1.22 (0.32, 4.55)	Excluded current users of at least 600 IU/d of supplemental Vit D or high-dose calcium therapy within the prior 6 m
Golubic 2018 Croatia [12]	NA metastatic colorectal cancer	69 51% total 46 m	during treatment Vit D3 (2000 IU/d)+standard treatment vs. standard treatment	1.01 (0.39, 2.61)	1.11 (0.69, 1.77)[a]	NA	NA	Not specified
**Observational Cohort Studies**								
Poole 2013 US+China [18]	After Breast Cancer Pooling Project breast cancers (I-III)	NA 0% NA	1-5 y after diagnosis users vs. non-users	0.95 (0.72, 1.24)	NA	0.97 (0.68, 1.38)	0.92 (0.62, 1.35)	Not specified
Ambrosone 2019 Mix	DELCaP	NA 0%	within 6 m (during chemotherapy)	1.05 (0.66, 1.65)	1.19 (0.81, 1.74)	NA	NA	Excluded users taking Vit D supplement before chemotherapy

[15]	breast cancer	median 8.1 y	users vs. non-users					
Madden 2018 Ireland [17]	NCRI invasive breast cancer(I-III)	NA 0% NA	within 6m & after 6m after diagnosis users vs. non-users	0.86 (0.72, 1.01)	NA	0.80 (0.64, .0.99)	NA	Excluded current users of taking Vit D supplement in the y prior to cancer diagnosis
Zeichner 2014 US [20]	UM/SCCC HER2+ nonmetastatic breast cancer	53 0% median 29.5 m for DFS, 40.4 m for OS	during chemotherapy users vs. non-users	0.30 (0.07, 1.37)	0.36 (0.15, 0.88)	NA	NA	Not specified
Wang 2016 China [19]	NA esophageal cancer	NA 83% 2 y	after surgery during EC treatment and recovery phase users vs. non-users	0.80 (0.51, 1.24)	0.61 (0.38, 0.98)	NA	NA	Not specified
Choi 2014 US [16]	IWHS All cancers	78.9 0% mean 6.1y	1-17 y after diagnosis users vs. non-users	0.82 (0.63, 1.08)	NA	NA	NA	Not specified

Abbreviations: IU, international unit; d, day; m, month; wk, week; NA, not available; Vit, Vitamin; y, year(s)

[a] data was extracted from a meta-analysis publication (Vaughan-Shaw PG, Buijs LF, Blackmur JP, Theodoratou E, Zgaga L, Din FVN, et al. The effect of vitamin D supplementation on survival in patients with colorectal cancer: systematic review and meta-analysis of randomised controlled trials. British Journal of Cancer. 2020;123(11):1705-12.)

Table S4. Confounding Factors Considered in Observational Cohort Studies

**First author, Year, Country, Reference**	**Confounding factors adjusted for**
Poole 2013 US+China [18]	age at diagnosis, exercise, stage, treatment, BMI, menopausal status, smoking, multivitamin use, antioxidants use
Ambrosone 2019 Mix [15]	age, ER, PR, HER2, lymph node status, tumor size, grades 3 or 4 hematologic toxicity, BMI, exercise, smoking, alcohol consumption, multivitamin use
Madden 2018 Ireland [17]	age at diagnosis, smoking, comorbidity score, tumor stage, tumor grade, ER, PR, HER2, bisphosphonate in year prior and post diagnosis, chemotherapy, anti-oestrogen therapy in year post diagnosis, NSAID, statins and anti-diabetic medication use in the year prior to diagnosis
Zeichner 2014 US [20]	age at diagnosis, tumor size, metastatic lymph nodes, histologic grade, ER, BMI at the end of chemotherapy
Wang 2016 China [19]	sex, exercise, pathological type, squamous cell carcinoma, tumor length, T category, stage, lymph node metastasis, ratio of lymph node, treatment regimen

Choi<br>2014<br>US<br>[16]	age, energy intake, BMI, exercise, smoking, total comorbidity index, perceived general, health, history of diabetes, history of high blood pressure, cancer type, cancer stage, surgery, chemotherapy, number of cancers, current cancer treatment, years since cancer diagnosis, diet quality score

Abbreviations: BMI, body mass index; ER, estrogen receptor; PR, progesterone receptor; HER2, human epidermal growth factor receptor 2; NSAID, non-steroidal anti-inflammatory drugs.
